# Cutaneous leukocytoclastic vasculitis secondary to COVID‐19 infection: A case report

**DOI:** 10.1002/ccr3.3596

**Published:** 2020-12-18

**Authors:** Fariba Iraji, Hamid Galehdari, Amir Hossein Siadat, Safoura Bokaei Jazi

**Affiliations:** ^1^ Dermatology Department Isfahan University of Medical Sciences Isfahan Iran; ^2^ Faculty of Pharmaceutical Sciences Isfahan University of Medical Sciences Isfahan Iran

**Keywords:** COVID‐19, cutaneous vasculitis, leukocytoclastic, SARS‐CoV‐2

## Abstract

COVID‐19 is a novel disease that mostly affects the respiratory system but as the number of cases is rising significantly around the world, more extra‐respiratory manifestations are being reported among which are various dermatologic manifestations.

## Graphical abstract

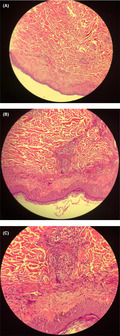

## 1 INTRODUCTION

The novel corona virus disease of 2019 (COVID‐19) is mostly known for its cardiac and pulmonary complications and it is often presented with respiratory symptoms. It is also associated with extra‐respiratory manifestations including dermatologic symptoms and complications. We report a case of cutaneous leukocytoclastic vasculitis presented with purpuric skin lesions in a patient with COVID‐19 infection.

The novel coronavirus disease of 2019 (COVID‐19) caused by severe acute respiratory syndrome coronavirus (SARS‐CoV‐2) was first identified in Wuhan, Hubei, China in December 2019 and resulted in a pandemic.^1^ As of July 2020, more than 12.9 million cases have been confirmed worldwide causing more than half a million deaths.^2^ The initial common manifestations of COVID‐19 included fever and respiratory symptoms such as cough, shortness of breath, and sputum production,^3^ but as the disease progressed rapidly more cases were reported with extra‐respiratory manifestations. Cardiac, gastrointestinal, renal, ocular, neurologic, and hepatic manifestations are among the common reported extra‐respiratory symptoms so far.^4^ Dermatologic manifestations and complications of COVID‐19 have also been reported in the recent literature and they include maculopapular rash, urticaria, vesicular rash, petechiae, perniosis, livedo racemosa and distal ischemia and necrosis.^5^, ^6^, ^7^, ^8^, ^9^, ^10^, ^11^ Here, we report a case of leukocytoclastic vasculitis secondary to COVID‐19 which presented with purpuric lesions on lower limbs.

## 2 CASE PRESENTATION

**FIGURE 1:**
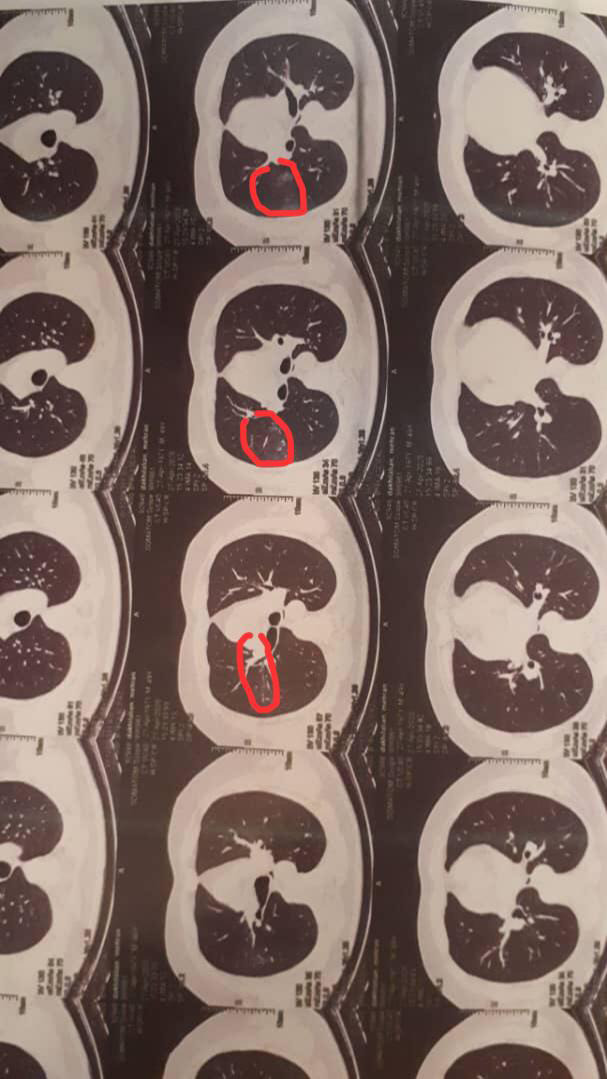
Ground glass opacity in CT scan

**FIGURE 2:**
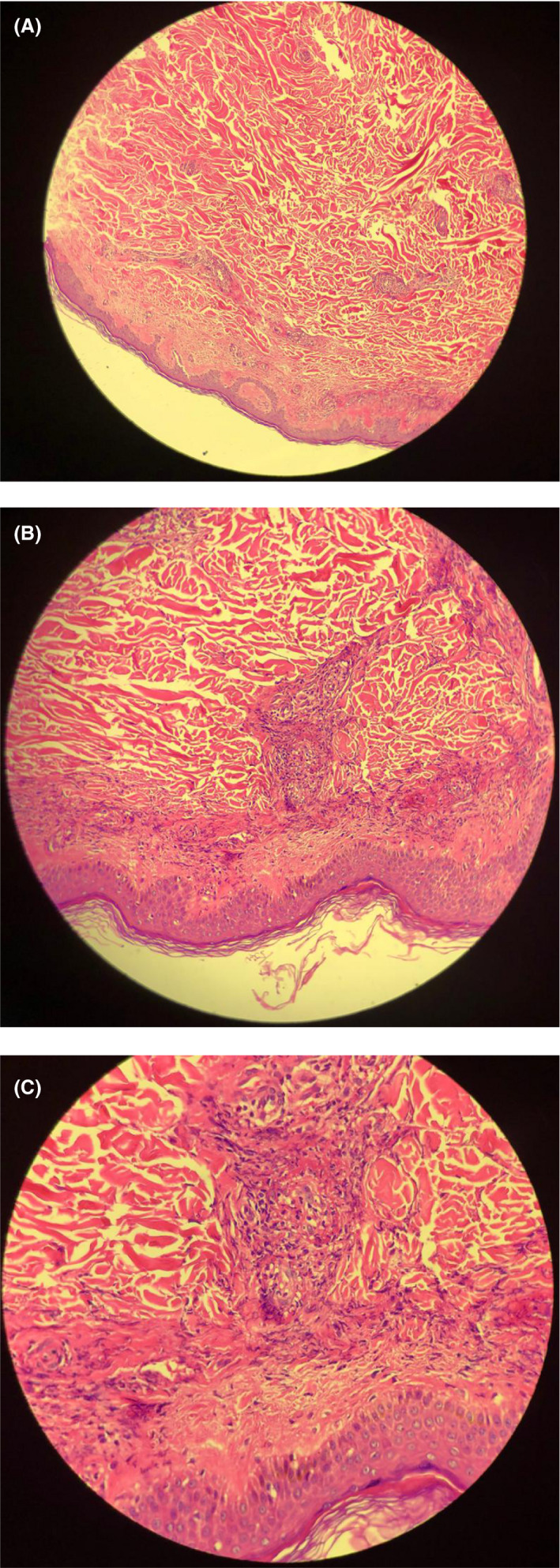
A, Mild hyperkeratosis of the epidermis (H&E ×100). B, Mild hyperkeratosis of the epidermis (H&E ×200). C, moderate neutrophilic infiltration, extravasated RBC, a few lymphocytes around superficial vessels (H&E ×400)

**FIGURE 3:**
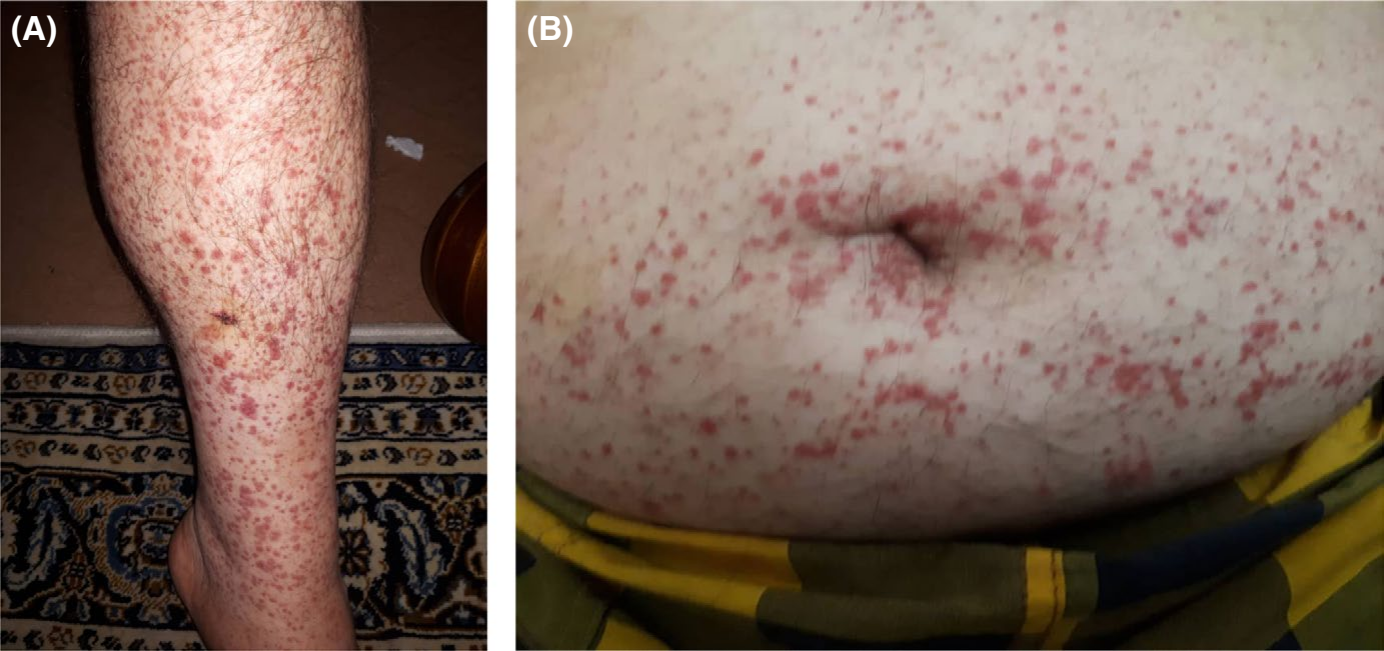
A, purpuric lesions on legs. B, purpuric lesions on abdomen

A 49‐year‐old otherwise healthy male physician presented to our dermatology department with the chief complaint of skin lesions on his lower limbs. He reported that he had started feeling unwell since April which was followed by aches and profound fatigue and lasted about 10 days. Subsequently, he started developing respiratory symptoms including cough and shortness of breath. He denied having other symptoms such as sore throat, fever, anosmia, or ageusia. On chest computed topography (CT), isolated patchy ground glass opacity accompanied with adjacent nodules with ground glass attenuation in middle zone of right lobe was seen that was suggestive for COVID‐19 (Figure 1). After consulting an internist and based on his symptoms and the imaging suggestive of COVID‐19 infection, he underwent treatment with hydroxychloroquine and azithromycin which led to his recovery although coughs persisted for a month. Two weeks later, a few rashes appeared on both of his legs that resembled insect bite reactions and progressed through the thighs and lower abdomen over a few days. Rashes rapidly increased in size and number over a short period of time causing a lot of discomfort for the patient in addition to cough and myalgia. He denied having any other symptoms including fever, abdominal pain, arthritis, or diarrhea. He also denied using any prescribed or herbal medications. He experienced two episodes of epistaxis during 24 hours. Full blood work up was done the following day and all were reported normal (Table 1). He was visited by a dermatologist and skin biopsy was taken. Histopathologic findings revealed mild hyperkeratosis of the epidermis, moderate neutrophilic infiltration, some extravasated red blood cells and a few lymphocytes around superficial and mid dermal vessels all consistent with leukocytoclastic vasculitis (Figure 2A,B,C). He was then referred to a rheumatologist for more assessments. Immunologic tests were requested and all reported within normal ranges (Table 1). By the time he presented to our dermatology department, skin lesion had worsened and became painful and pruritic. A more detailed history was taken from the patient. On physical examination, there were purpuric lesions on flexor and extensor parts of both legs and thighs and lower abdomen (Figure 3A,B). They were round in shape in different sizes and were completely palpable. No pustules, bullae, or urticaria was seen. Physical examinations of cardiopulmonary, musculoskeletal, and gastrointestinal systems were normal and cutaneous leukocytoclastic vasculitis was confirmed based on the findings of skin biopsy and the clinical presentation.

**TABLE 1:** Para clinical results

	Result	Normal range
WBC	7100/uL	4500‐11 000
Hb	14.9 g/dL	13‐17.5
Neutrophils	56%	40‐80
Lymphocytes	37.2%	20‐60
Platelet	260 000/uL	150 000‐450 000
FBS	97 mg/dL	70‐100
BUN	21 mg/dL	19‐44
Cr	1.08 mg/dL	0.7‐1.4
CRP	Negative	Negative
PT	13 s	12‐14
PTT	35 s	25‐38
Urinalysis	Normal	
IgA	2.7 g/L	0.7‐4
Cryoglobulin	2.15 IU/mL	<5.0
C3	1.5 g/L	0.8‐1.85
C4	0.37 g/L	0.1‐0.4
ANCA‐P	0.25 U/mL	<12
ANCA‐C	0.20 U/mL	<12
ANA	0.18 index	<0.9
Anti ds‐DNA	2.7 iu/mL	<16

## 3 DISCUSSION

COVID‐19 presents with many extra‐respiratory manifestations in addition to respiratory symptoms and fever.^12^ An increasing number of dermatologic manifestations are being attributed to COVID‐19 as the number of cases are rapidly progressing.^13^ Racalcati et al^6^ reported that 20.4% of 88 confirmed cases of COVID‐19 had developed cutaneous manifestations which mostly presented with erythematous rash. According to the recent literature, the most common form of cutaneous involvement is a generalized macular or maculopapular rash (36.1%) followed by papulovesicular rash (34.7%) and urticaria (9.7%). The majority of lesions appeared on the trunk and extremities. Lesions had developed prior to COVID‐19 diagnosis, at the onset of symptoms or up to 2 weeks after diagnosis.^14^

Also, in our case report, the patient developed skin lesions 2 weeks after the diagnosis of COVID‐19 and they were located on the lower limbs and trunk with histopathologic findings suggesting cutaneous leukocytoclastic vasculitis. Leukocytoclastic vasculitis (LCV) is characterized by leukocytoclasis, which refers to vascular damage caused by infiltration of neutrophils.^15^ It is a rare condition that affects 30 million people each year. The typical clinical presentation of LCV is palpable purpura on the lower extremities that can be associated with pain and pruritus. Other cutaneous presentations of LCV include ulcers, pustules, and vesicles. LCV may also be associated with systemic symptoms when internal organs such as gastrointestinal tract, kidneys, and joints are affected by the condition. These symptoms include arthralgia, arthritis, myalgia, abdominal pain, fever, diarrhea, melena, cough, hematochezia, and many more.^16^ Almost half of LCV cases are idiopathic and others occur secondary to underlying infections, connective tissue disorders, malignancy, and medications. The exact mechanism of LCV is still unknown but immune complex deposition plays an important role in the pathophysiology of LCV.^17^, ^18^, ^19^ The diagnosis of LCV is confirmed by skin biopsy. The presence of vascular and perivascular infiltration of polymorphonuclear leukocytes with formation of nuclear dust (leukocytoclasis), extravasation of erythrocytes, and fibrinoid necrosis of the vessel walls are typical histopathologic findings of LCV.^20^

The pathophysiology of dermatological manifestations of COVID‐19 still remains uncertain, however some theories have been suggested in recent studies.^14^ The etiologic agent of COVID‐19 is an RNA virus that enters cells through the angiotensin‐converting enzyme 2 (ACE2) receptor which is found in lungs, intestines, vasculature, cardiac, and nervous systems,^21^, ^22^ being responsible for multiple organ damages. Recent studies suggest that ACE2 is also located in the skin which may explain some of the dermatologic manifestations of COVID‐19.^23^ Mogro et al reported pauci‐inflammatory thrombogenic vasculopathy with deposition of C5b‐9 and C4d as well as co‐localization of these with COVID‐19 spike glycoproteins.^24^ We report a case of cutaneous leukocytoclastic vasculitis in a patient with COVID‐19 with typical presentation and histopathology and normal complement levels. Also, lymphocytic vasculitis presenting with skin lesions on toes and feet have been reported in COVID‐19 patients who were mostly children and adolescents.^25^, ^26^ It is still unclear whether these cutaneous symptoms are secondary consequences of respiratory infection or the primary involvement of the skin caused by the infection. Further studies should validate and elucidate the mechanism of such conditions. Physicians should update their knowledge about extra‐respiratory and less common manifestations of COVID‐19 to get a better understanding of the disease and its evolving path.

## CONFLICT OF INTEREST

We have no conflicts of interest to declare.

## AUTHOR CONTRIBUTIONS

All the authors listed in the manuscript have participated actively and equally in presenting the case and providing the final version of the manuscript.

## ETHICAL APPROVAL

This case report was approved by the bioethics committee of Isfahan University of Medical Sciences. Written informed consent was taken from the patient.

## Data Availability

No datasets were generated or analyzed during this case report.
